# Dystrophic Epidermolysis Bullosa (DEB): How Can Pregnancy Alter the Course of This Rare Disease? An Updated Literature Review on Obstetrical Management with an Additional Italian Experience

**DOI:** 10.3390/diseases12050104

**Published:** 2024-05-15

**Authors:** Antonella Vimercati, Gerardo Cazzato, Lucia Lospalluti, Stefania Foligno, Cristina Taliento, Katarzyna Beata Trojanowska, Ettore Cicinelli, Domenico Bonamonte, Dario Caliandro, Amerigo Vitagliano, Pierpaolo Nicolì

**Affiliations:** 1 Unit of Obstetrics and Gynecology, Department of Interdisciplinary Medicine (DIM), University of Bari “Aldo Moro”, Policlinico of Bari, Piazza Giulio Cesare 11, 70124 Bari, Italy; 2 Department of Emergency and Organ Transplantation (DETO), Section of Pathology, University of Bari “Aldo Moro”, Policlinico of Bari, Piazza Giulio Cesare 11, 70124 Bari, Italy; 3 Department of Biomedical Sciences and Human Oncology (DIMO), Section of Dermatology and Venereology, University of Bari “Aldo Moro”, Policlinico of Bari, Piazza Giulio Cesare 11, 70124 Bari, Italy; 4 Department of Medical Sciences, Obstetrics and Gynecology Unit, University Hospital “Sant’Anna”, 44121 Ferrara, Italy; 5 Section II of Anesthesia and Resuscitation, Policlinico of Bari, 70121 Bari, Italy; 6 Pelvic Floor Center, Department of Gynaecology and Obstetrics, Pia Fondazione “Card. G. Panico”, 73039 Tricase, Italy

**Keywords:** dystrophic epidermolysis bullosa, pregnancy, delivery, management

## Abstract

Epidermolysis Bullosa (EB) is an extremely rare and disabling inherited genetic skin disease with a predisposition to develop bullous lesions on the skin and inner mucous membranes, occurring after mild friction or trauma, or even spontaneously. Within the spectrum of EB forms, dystrophic EB (DEB) represents the most intriguing and challenging in terms of clinical management, especially with regard to pregnancy, due to the highly disabling and life-threatening phenotype. Disappointingly, in the literature little focus has been directed towards pregnancy and childbirth in DEB patients, resulting in a lack of sound evidence and guidance for patients themselves and clinicians. The current study aims to contribute to the DEB literature with an updated summary of the existing evidence regarding the obstetrical and anesthesiological management of this rare disease. Furthermore, this literature review sought to answer the question of whether, and if so, in which way, the pregnancy condition may alter the course of the underlying dermatologic skin disease. Having all this information is indispensable when counseling a patient with DEB who desires a child or is expecting one. Finally, we reported own experience with a pregnant woman with a recessive DEB whom we recently managed, with a favorable outcome.

## 1. Introduction

Epidermolysis bullosa (EB) represents a cluster of rare and disabling inherited genetic diseases with a predisposition to develop bullous lesions on the skin and inner mucous membranes, occurring after mild friction or trauma, or even spontaneously [1]. Historically, these patients’ cutaneous brittleness has been likened to the proverbial fragility of a butterfly’s wings, hence the ancient epithet “*butterfly baby syndrome*”.

It is estimated that worldwide EB affects 1 in every 17,000 births and a total number of about 500,000 people, regardless of race, sex, or ethnicity. Self-evidently, these figures shrink when considering Italy alone, with an estimated 1 affected patient in every 82,000 births and an aggregate number of about 1,500 EB sufferers expected in our country [2].

Pathogenetically, it is widely recognized that EB arises as an effect of inherited abnormalities of single or multiple anchor proteins that form the interconnecting network from the epidermis to the underlying dermis. Based on the mechanism involved at a molecular level and, therefore, the level in the skin where blisters occur, three main types of EB have been described historically: (i) EB simplex; (ii) junctional EB (JEB); and (iii) dystrophic EB (DEB) [1]. Actually, nowadays experts tend to include even the extremely rare Kindler EB within the spectrum of main EB forms [1]. Clinically, these forms of EB vary in the degree of skin involvement and, as a consequence, in prognosis and disability throughout life. Regarding the first three forms, whether the simplex form is usually the most common and the least severe variant (with affected patients experiencing a near-normal life), and the junctional form is the least frequent and deadliest among them, unquestionably DEB is the most intriguing and challenging in terms of clinical management.

DEB results from mutations in the COL7A1 gene that codes for type VII collagen [3] and leads to blistering in the dermis of the skin and other epithelial surfaces. Although DEB can be inherited as an autosomal dominant (DDEB) or recessive trait (RDEB), RDEB accounts for the majority of sporadic cases of DEB, with an estimated prevalence, in terms of cases per million population, of 1.35 in the Unites States, based on the National EB Registry [4], and 1.5–2.1 in Japan, based on data from the Japanese Study Group for Rare Intractable Skin Diseases [5]. However, on the whole reliable data about RDEB prevalence are negligible. While DDEB usually has a more subdued presentation with blisters primarily affecting the friction sites, RDEB often results in a severe and highly disabling phenotype with blisters that are widely spread throughout the body and slow to heal. Furthermore, the healing process typically results in a characteristic atrophic scarring that can lead to nail loss, pseudosyndactyly and contractures of wrist, elbow, knee and ankle joints. Other sites frequently involved are teeth, mouth and esophagus, resulting in teeth malformation, microstomia and esophageal strictures; the subsequent difficulty in mastication and swallowing leads to malnutrition, anemia, growth delay and weight loss [6]. Intuitively, all these clinical features may remarkably complicate the management of pregnancy in patients suffering from DEB, especially with regard to delivery, both from an obstetrical and anesthesiological perspective. However, in the literature there are only a few reports of patients with DEB becoming pregnant, thus resulting in a scarcity of evidence addressing this important issue. A related and equally important question to answer is whether, on the contrary, pregnancy itself can alter the course of the underlying dermatologic skin disease, by exacerbating or mitigating it. Having all this information is crucial when counseling patients with DEB who are pregnant or planning a family. Hence, the aim of the current review article is to fill this gap by collating the negligible evidence reported in the literature and summarizing the difficulties of managing such women during pregnancy, delivery and postnatal period. We decided to conclude the summary by reporting our own experience with a pregnant woman with RDEB whom we recently managed, with a favorable outcome.

## 2. Antenatal Care

### 2.1. Prenatal Genetic Testing

As a rule of thumb, a clinical genetics consultation should be offered to both male and female patients with EB considering pregnancy or who have found out they are expecting a child, in order to estimate and explain the risks of inheritance [7].

Depending on the inheritance pattern, prenatal diagnosis could be performed at the beginning of the pregnancy by using molecular genetic methods on chorionic villus samples or, later, on the amniotic fluid. However, prenatal testing is not usually offered when one partner has RDEB as, though every child will be a carrier, the risk of having an affected child is considerably lower as compared with the risk of miscarriage from chorionic villous sampling (1 in 700 vs. 0.5% to 2%, respectively) [8]. Similarly, carrier screening of an unaffected and unrelated partner may be offered depending on the individual situation and national regulations, after genetic counselling [9]; however, it is usually limited to exceptional cases, such as blood-related partners or partners with known EB in the family.

Pregnancy in women with DEB has been reported in the literature (Table 1), but only in a few cases is reference made to prenatal genetic investigations [10,11,12]. Büscher et al. [10] reported a case of a pregnant woman with RBED and a healthy partner who requested genetic counseling during which the couple was informed that the risk of DEB occurrence in offspring was less than 1% (no consanguinity, no known EB in the husband’s family). In Bianca and colleagues’ work [11], no DNA mutations were found in the husband of a RDEB patient; therefore, prenatal diagnosis was not considered. Boria and coworkers [12] described the case of a 40-year-old woman with severe generalized RDEB (due to two mutations in heterozygosity in exons 34 and 80 of the COL7A1 gene) married to a man suffering from *retinitis pigmentosa*; she underwent amniocentesis at 15 weeks’ gestation and the presence of EB in the fetus was excluded. Finally, Colgrove et al. [13] and Intong et al. [14] described cases of mothers suffering from DDEB who gave birth to affected children, but none of them referred to any prenatal diagnostic test.

### 2.2. Maternal-Fetal Clinical Monitoring

Aiming to optimize pregnancy management, pregnant patients with complex forms of EB, such as DEB, should be followed by a multidisciplinary team (MDT) consisting of an obstetrician, midwife, anesthetist, dermatologist, psychologist and nutritionist [8,13,14,15,16].

In particular, RDEB may be associated with several diseases related to the underlying dermatologic skin condition that may complicate the management of pregnancy, such as renal impairment (for example, renal failure or nephrotic syndrome due to exacerbations) [17,18]; hence, a close antenatal monitoring of the pregnant women suffering from DEB with complete blood exams is desirable. Furthermore, as the available literature shows (Table 1), the condition of anemia often afflicting DEB patients who have difficulty feeding themselves tends to worsen during pregnancy [10,12,16,19]. Sometimes, because of the vulnerability of the esophagus, oral iron substitution may be impracticable [10]; hence, based on the degree of anemia, intravenous administration of iron [12,19] or blood transfusions [16] have to be given in order to maintain the patient’s hemoglobin levels. Anemia was associated with, respectively, hypothyroidism and vitamin D deficiency in two cases reported by Boria et al. [12]. Noteworthy, Araújo et al. [15] reported a case of squamous cell cancer (SCC) that arose early in the pregnancy and led to amputation of the right hand, while Lopes and coworkers [20] described a case of SCC that arose before conception and progressed during pregnancy.

On the whole, the collated literature (Table 1) seems to suggest that patients with DEB do not have any additional prenatal complications unrelated to DEB as compared to the general population. Pregnancy was complicated by pre-eclampsia and gestational diabetes mellitus (GDM) only in one report [21] out of fifteen and two reports [10,12] out of fifteen, respectively. In one case [10], GDM was accompanied by polyhydramnios. Additionally, regular ultrasound checks during the pregnancy revealed fetal growth restriction (FGR) in three cases [11,12,22], one of which [22] associated with anhydramnios, but there is still no firm evidence of association between EB and FGR. Finally, in none of the examined cases of DEB (Table 1) was there any reference to specific ultrasound signs suggestive of EB. Although ultrasound is not used for prenatal diagnosis of EB [9], some cases of fetal EB primarily suspected by ultrasound are described in the literature. EB was first hypothesized [23] on prenatal ultrasounds in a couple at risk due to the presence in the amniotic fluid of “snowflakes” (namely echogenic particles). Other related sonographic findings reported in the literature include enlarged stomach and polyhydramnios [24,25] (due to frequent association with pyloric atresia), deformed external ears and contracted fisted hand [24], shortening of long bones [26], complete chorioamniotic membrane separation [27] and localized skin denudation [28]. However, all these references are sporadic and lack scientific solidity, so a definite prenatal ultrasound diagnosis of EB still remains a fundamentally unresolved challenge. Nevertheless, looking for these signs during normal ultrasound checks may be considered appropriate and good clinical practice, even though genetic counselling has ruled out the possibility of EB transmission to the fetus. In addition, as a good rule, in case of termination of pregnancy or intrauterine fetal death, the diagnosis of such rare and complex fetal abnormalities that are suspected via ultrasound should always be confirmed by histological/immunohistochemical examinations during autopsy [29,30].

All the remaining standard antenatal investigations for pregnancy should be performed in DEB patients, but with a few precautions, e.g., (i) antenatal blood pressure monitoring should be executed with well-padded blood pressure cuffs, taking care to avoid frictional or shearing forces; (ii) ultrasound exams require generous lubrication prior to assessment, gentle pressure and the selection of the smallest probe in case of vaginal exploration; (iii) cardiotocography should be used only when clinically indicated, as it results in a high risk of blistering.

## 3. Course of DEB during Pregnancy

Women with severe forms of EB, such as JEB or DEB, often do not consider having a child because of difficulties in finding a partner with whom they want or can have a child. Worth mentioning, the fear of DEB patients that their underlying skin condition will worsen during pregnancy may be another main reason why they do not consider having a child, as well as the possibility of normal sexual activity. Actually, a few cases of worsening of the disease during gestation are described in the literature (Table 1). As reported by Hanafusa et al. [19], in two patients with moderate RDEB the number of skin ulcers around their lower abdomen increased as it became distended in pregnancy, even though no genital mucosal ulcers appeared. In addition, Ozakaya and colleagues [22] reported a case of RDEB with worsening of the disease at term of pregnancy, with the appearance of widespread skin erosions, including the vulval region and vaginal mucosa, compatible with genital involvement of DEB. Finally, in Boria’s RDEB patient [12] skin lesions tended to become more infected in pregnancy. However, notwithstanding these few reports, overall it seems unlikely that DEB itself worsens as a result of pregnancy (Table 1), with clinical stability of the skin disease reported in most cases within the available literature [10,11,13,14,31]. Worth mentioning, Intong et al. [14] even reported the case of a DEB patient in whom the skin condition improved during pregnancy: there is no solid scientific basis to explain this phenomenon, hence we resort to general knowledge that has been widely demonstrated and accepted by the international scientific community. Indeed, it is well known that pregnancy represents a period of relative immune tolerance and “physiological” immunosuppression to prevent the mother’s immune system from attacking the fetal–placental structures, bearing half the paternal genetic heritage and half the maternal genetic heritage [32]. Therefore, it could be plausible that an immune shift towards greater immunological tolerance was the basis for the clinical improvement of the lesions in the patient in question, corroborating the knowledge already acquired regarding a general non-worsening of DEB during gestation. Also, the trend of oncological lesions in pregnant DEB patients (*Paragraph 2.2*) is worthy of further investigation because it has the potential to shed light on certain immunological mechanisms during pregnancy.

## 4. Delivery

Intuitively, the main complications for pregnant patients with DEB can arise at the time of delivery.

Which is the right *mode* of delivery for patients with DEB is certainly one of the most difficult knots to unravel. Although at first glance the characteristics of this rare disease may suggest that a caesarean section (CS) may be the safest way to go, our literature search (Table 1) has shown that vaginal delivery (VD) is not a contraindication in patients affected by DEB and can take place successfully: overall, VDs without any complications in DEB patients have been described in five studies [8,10,14,19,31] out of fifteen, after mediolateral episiotomy in some cases [10,14,19] and with vaginal wall tears in others [8,14,31]. As for the skin wound healing (Table 1), after VD both episiotomy and perineal tears have been shown to heal well in women with DEB [8,10,14,19,31]. Therefore, standard obstetrical management of the episiotomy/tear site, including sutures, can be recommended in these patients [10,14]. Moreover, no cases of vaginal stenosis after childbirth have been reported in patients with DEB; rather, patency of the birth canal after delivery has been confirmed in four reports of RDEB patients who delivered more than one child vaginally [10,14,19,31]. However, though the reported cases went smoothly, this method cannot be considered completely complication-free. In theory, blistering of the lower back, buttocks, legs and elbows may occur during prolonged VD, based on the patient’s positions during labor; in addition, VD may be associated with vaginal mucosa blistering and scarring, especially if the application of forceps or a vacuum extractor is necessary during childbirth. Therefore, mainly owing to fear and anxiety regarding possible trauma to the birth canal, in some cases there may be a strong maternal preference for elective CS [8,13,16]. Elective or emergency CS revealed itself as being mandatory within six studies [11,12,15,17,21,22], due to (i) genital mucous lesions in two cases [11,15]; (ii) vaginal stenosis in one case [12]; and (iii) obstetrical indications within the remaining three studies [17,21,22]. Finally, indications for CS were not provided in three cases [14,20,33]. Overall, data available in the literature seem to encourage the vaginal route for delivery whenever possible.

Regarding the *time* of delivery, there seems to be no doubt that these pregnancies usually can come to term uneventfully, as do those of DEB-unaffected patients. In almost all reviewed cases (Table 1) childbirth occurred at term of pregnancy [8,10,12,13,16,17,19,22,31,33], with only a few exceptions of near-term delivery, owing to obstetrical [11,21] or non-obstetrical [15] reasons. In two cases [12,20], reasons for near-term elective CS were not reported. 

## 5. Anesthesia

An additional reason for preferring VD to the surgical route, whenever indicated and feasible, is related to the airway management, which may often be challenging in DEB patients. Microstomia, poor dentition and esophageal stenosis frequently present in severe forms of DEB can make intubation an extremely difficult procedure, also possibly resulting in life-threatening bullae of lips, tongue, pharynx and epiglottis [21,34]. Therefore, in the literature regional anesthesia (RA) has widely been preferred to the general one for patients with DEB [8,11,13,15,16,17,33], both in case of emergency and elective CSs (Table 1). RA is not a complication-free procedure, due to the possible formation of new bullae at the puncture site, although no cases of skin complications have been reported as a direct result of this anesthetic method. (Table 1). Furthermore, an a priori in-depth inspection of the puncture site is always required, since a sign of infection at this level may be a contraindication for RA. Indeed, in one case [21] physicians considered RA contraindicated because of the presence of infected ulcerated bullae on the skin over the lumbosacral spine, thus opting for general anesthesia. One exception to this general rule has been reported by Bolt and colleagues [16]: despite the presence at the site of needle puncture of an infected blister that tested positive for methicillin-resistant Staphylococcus aureus (MRSA) and the resulting increased risk of developing bacterial meningitis from a RA, this latter was thought to carry an overall lower risk than a general anesthesia.

## 6. Postnatal Care

The RDEB patient within the study by Lopes et al. [20] died 6 months after delivery due to neoplastic progression of an invasive squamous cell cancer (SCC) aroused before pregnancy. As reported above (*Paragraph 4*), after VD both episiotomies and perineal tears tend to heal well in women with DEB [8,10,14,19,31]. On the whole, the same applies to CS wounds (both in case of elective and emergency CSs) [8,11,12,13,15,16,22,33], with only a few cases of scar blistering described [11,16]. Non-adherent dressing is usually utilized for both types of wounds.

With the exception of one case of eclampsia [21] and one case of post-partum hemorrhage (PPE) [19], most of the reviewed studies reported no complications after delivery [8,10,11,12,13,15,16,17,22,31,33]. No data about the postnatal course is reported by Intong and colleagues [14]. Thus, overall our literature search (Table 1) depicts the absence of additional post-natal complications unrelated to DEB as compared to non-affected patients [35].

Breast-feeding has been described in women with DEB [8,10,14,16,19], although a high rate of blistering around the nipples has been reported [8,10,14,16], leading to discontinuation in some cases [8,10,14]. In other cases, patients have not breastfed because of blisters around the nipples [12,19] or to prevent the formation of blisters during suckling, either voluntarily [13] or on physicians’ advice [19,31]. Overall, the collated evidence (Table 1) seems to encourage DEB mothers to breastfeed when possible. Patients should be educated to position the neonate’s mouth correctly around the part of the areola that has tougher skin and well-lubricated nipple shields should be offered to all mothers in order to reduce bullae formation [8,16].

Table 1 provides an overview of the pregnancies reported in the literature in DEB patients, together with relative course, management and outcomes. We have included an additional case that we have recently managed, previously unpublished and described below.

**Table 1:** Overview of the pregnancies in DEB patients reported in the literature, together with relative management and outcomes. An additional case, recently managed by us and previously unpublished, has been added.

First Author (Year) Study Type	Number of Cases (Age, in Years)	Type of DEB (Severity)	Parity	Trend of Blistering during Pregnancy	Antenatal Complications	Gestation at Delivery	Mode of Delivery	Indication for Mode of Delivery	Anesthesia	Skin Wound Healing	Clinical Complications	Breast Feeding
Berryhill et al. (1978) [21] CR	1 (35)	ND (Severe)	ND	ND	Preeclampsia	34.6	Emergency CS	SROM, breech presentation	General (spinal/epidural anesthesia contraindicated due to infected lumbosacral blisters)	ND	Eclampsia	ND
Broster et al. (1987) [17] CR	1 (17)	DDEB (Moderate)	First	ND	None	Term	Emergency CS	Primary cephalo-pelvic disproportion	Epidural	ND	None	ND
Büscher et al. (1997) [10] CR	1 (24)	RDEB (severe)	First	Stable	None	Term	Vaginal—after mediolateral episiotomy	/	Local	Uncomplicated	None	Yes, but discontinued because of blister formation around the nipples
	1 (24)—same patient, second pregnancy	RDEB (severe)	Second	Stable	Anemia; GDM; polyhydramnios	Term	Vaginal—after mediolateral episiotomy	/	ND	Uncomplicated	None	ND
Bianca et al. (2003) [11] CR	1 (ND)	RDEB (severe)	First	Stable	FGR	36.0	Emergency CS	Genital mucous lesions; FGR; PPROM with pre-term labour	Epidural	A blister around CS scar	None	ND
Baloch et al. (2008) [8] CR	1 (29)	RDEB (severe)	First	ND	None	40.0	Vaginal—with small posterior vaginal wall tear (not sutured)	/	None	Uncomplicated	None	Uncomplicated
	1 (33)	RDEB (severe)	First	ND	None	39.1	Emergency CS	SROM; patient’s request	Spinal	Uncomplicated	None	Yes, but discontinued because of blister formation around the nipples
Bolt et al. (2009) [16] CR + literature review	1 (25)	RDEB (severe)	Second	ND	Anemia; MRSA-infected lumbosacral blisters	38.2	Elective CS	Patient’s request	Spinal (risks of general anesthesia deemed greater than spinal at infected site)	Few blisters at CS scar	None	Yes, and continued despite blister formation around the nipples
Choi et al. (2011) [31] CR	1 (ND)	RDEB (severe)	3 pregnancies	Stable	None	Term in all pregnancies	Vaginal—with vaginal wall tear (sutured)	/	ND	Uncomplicated	None	No, to prevent blistering around the nipples (doctors’ choice)
	1 (ND)	RDEB (severe)	5 pregnancies	Stable	None	Term in all pregnancies	Vaginal	/	ND	None	None	ND
Hanafusa et al. (2012) [19] CR	1 (30)	RDEB (severe)	First	Worsened (increase in skin ulcers around lower abdomen)	Moderate anemia	Term	Vaginal—after mediolateral episiotomy	/	ND	Uncomplicated	None	No, to prevent blistering around the nipples (doctors’ choice)
	1 (27)	RDEB (severe)	2 pregnancies	ND	First pregnancy: threatened miscarriage. Second pregnancy: slight anemia	Term in all pregnancies	Vaginal—after mediolateral episiotomy	/	ND	Uncomplicated	First delivery complicated by PPE	Yes
	1 (21)	RDEB (severe)	First	Worsened (increase in skin ulcers around lower abdomen)	Moderate anemia	39.6	Vaginal	/	ND	Uncomplicated	None	No, because of blisters around the nipples
Ozkaya et al. (2012) [22] CR	1 (26)	RDEB (ND)	First	Worsened after 34 weeks (widespread skin erosions, including the vulval region and vaginal mucosa)	Anhydramnios; FGR	40.0	Emergent CS	Skin erosions on vulval region and vaginal mucosa; non-reassuring fetal status at admission for initial labor	ND	Uncomplicated	None	ND
Colgrove et al. (2014) [13] CR + literature review	1 (19)	ADDEB (mild)	Fifth (but the first to proceed after four abortions)	Stable	None	39.0	Elective CS	Patient’s request	Spinal	Uncomplicated	None	No, to prevent blistering around the nipples (doctors’ choice)
Turmo-Tejera et al. (2014) [33] CR	1 (28)	RDEB (severe)	First	ND	ND	37.0	Elective CS	ND	Spinal	Uncomplicated	None	ND
Araújo et al. (2017) [15] CR	1 (26)	RDEB (severe)	First	ND	SCC in early pregnancy requiring amputation of right hand; anemia	36.0	Elective CS	Lesions in the vaginal canal; appearance of a right axillary swelling requiring urgent investigation (suspected metastasis)	Spinal	Uncomplicated	None	ND
Intong et al. (2017) [14] Retrospective study	12 patients (ND)	DDEB (ND)	ND	All stable	ND	ND	26 vaginal; 4 emergency CS	ND	ND	As for vaginal, all reported good healing of their episiotomy and tears, where occurring	ND	Yes
	3 patients (ND)	RDEB (ND)	ND	2 stable; 1 improved	ND	ND	8 vaginal; 1 elective CS	ND	ND	As for vaginal, all reported good healing of their episiotomy and tears, where occurring	ND	Yes, but 1 discontinued because of blister formation around the nipples
Boria et al. (2019) [12] CR	1 (40)	RDEB (severe)	First	Worsened (infection of skin lesions)	Hypothyroidism; iron-deficiency anemia; GDM; FGR	37.4	Elective CS	Vaginal stenosis	ND	Uncomplicated	None	No, because of blisters around the nipples
	1 (25)	RDEB (moderate)	Second	ND	Iron-deficiency anemia; vitamin D deficiency	36.2	Elective CS	Previous CS; vaginal stenosis	ND	Uncomplicated	None	Yes
Lopes et al. (2020) [20] CR	1 (25)	RDEB (severe)	Second (but the first to proceed after one abortion)	SCC worsened	Invasive SCC aroused before pregnancy	36.0	CS	ND	ND	ND	Death 6 months after delivery due to neoplastic progression	ND
Vimercati et al. (2024)	1 (36)	RDEB (severe)	First	Improved—with rebound effect after childbirth	Anemia; FGR	36.1	Emergency CS	Initial labor; breech presentation	Combined, peridural and spinal	Uncomplicated	None	Yes, but discontinued because of blister formation around the nipples

CR = case report; CS = cesarean section; FGR = fetal growth restriction; GDM = gestational diabetes mellitus; ND = No data; PPE = postpartum hemorrhage; PPROM = premature preterm rupture of membranes; SROM = spontaneous rupture of membranes.

## 7. An Italian Experience

At 14.6 weeks of gestation, a 36-year-old female of Caucasian ethnicity with gravida 0 para 0 abortion 0 (G0P0Ab0) and suffering from a severe form of RDEB was referred to our High-Risk Pregnancy Unit, Department of Obstetrics, University Hospital of Bari, Puglia, Italy. The patient was not news to the dermatologists of the same hospital, who had followed her since birth before referring her to us once she became pregnant.

Upon the reconstruction of the family tree, no other members of the patient’s family were found to have EB or other established or presumed dermatological or genetic disorders; furthermore, although they both came from the same area in Puglia (southern Italy), the patient’s parents were not blood relatives. Nonetheless, the woman primarily received the diagnosis of DEB in 1994, based on electron microscopy and a subsequent genetic confirmation. The genetic analysis identified a double heterozygous mutation, compatible with a recessive transmission: a *c.6528del* mutation was found on exon 80 of the COL7A1 gene and a *c.8304+1G>C* mutation was detected on exon 111 of the same gene. 

Over the course of her life, the woman had developed many cutaneous blisters and scars and mild pseudosyndactyly of hands and feet, with partial loss of interdigital spaces and nails; furthermore, in adulthood she had undergone two local excisions for invasive SCCs located on the posterior surface of the right thigh and the sole of the right foot, respectively. Also, she reported difficulty swallowing solid foods and moderate dysphagia, even though the esophageal involvement never required any dilatations.

As for pregnancy, the patient had conceived spontaneously. Her anamnesis was silent for drug use, alcohol drinking, smoking or pregnancy infection. At admission at our Unit, a low BMI of 17.5 Kg/m^2^ and moderate anemia (9.1 g/dL) were highlighted; the remaining blood values were substantially normal. 

Although the patient’s husband reported a good health status and denied any dermatological problems in his bloodline and consanguinity with his partner, we asked him to undergo genetic testing in order to estimate the risk of DEB parental transmission to offspring. The results showed no pathogenic or likely pathogenic variants in the COL7A1 gene, ruling out the carrier status and enabling us to avoid performing invasive prenatal diagnosis procedure.

The first-trimester ultrasound screening could not be performed due to the advanced gestation period at the first visit. However, the non-invasive prenatal test (NIPT) revealed no major aneuploidies (on chromosomes 13, 18 and 21). Also, the second-trimester ultrasound anatomy scanning was normal and there were no ultrasound signs suggestive of EB.

Interestingly, during ongoing antenatal monitoring DEB revealed itself as being significantly attenuated: dysphagia progressively reduced and, overall, the number of skin blisters saw a sizeable decline, even though skin lesions healed more slowly and became infected more frequently. At 23.6 weeks of gestation, the patient’s haemoglobin level dropped to 7.7 g/dL and, as a result, a ferric carboxymaltose injection was given, thus bringing the hemoglobin level back to its starting level. Finally, despite normal blood exams, some episodes of itching on the abdomen and thighs were reported in the last months of pregnancy; fortunately, they were not followed by the onset of skin lesion.

A MDT, including obstetricians, dermatologists and obstetrical anaesthetists, extensively informed the patient about all the available options for delivering. Therefore, also due to the lack of vaginal stenosis and lesions in the genital region and vagina, it was agreed early in pregnancy that a VD would be the safest option. However, at 36.1 weeks of gestation, the patient was admitted for incipient labor, but the fetus had a breech presentation, thus precluding a vaginal birth; therefore, it was decided for an emergency CS. Of note, the admission ultrasound examination revealed FGR, with adequate amniotic fluid and normal Doppler velocimetry.

Before delivery, the patient underwent an in-depth anesthesiological counselling and an ASA 4 score was finally assigned. Noticeably, airway management proved to be extremely challenging, identifying the following clinical factors: (i) the highest score (4) on the Mallampati scale, a widely known classification used in anesthesia to predict the difficulty of oro-tracheal intubation, (ii) mouth opening less than 1 cm (severe microstomy), (iii) severely reduced lingual movements (ankyloglossia) as a consequence of DEB-related tenacious adherence of the tongue to the floor of the mouth, (iv) mobile molar teeth and (v) difficulty swallowing. Additionally, at inspection the skin overlying the lumbar spine did not show active lesions, but only a few scars. Therefore, in agreement with the patient, we decided to perform a RA, precisely a combination of peridural and spinal anesthesia.

Before the commencement of surgery, the operating table was padded with an anti-decubitus mattress and the blood pressure cuff with cotton wool; moreover, non-adhesive ECG patches were used for the standard intraoperative monitoring. A venous access (with an 18G needle cannula) was easily established on the left forearm (radial vein) and secured with a non-adhesive dressing (Mepilex plaster). Crystalloids and pre-anesthetic drugs were firstly administered intravenously. Subsequently, in the sitting position and after dabbing the skin of the back with a moist Iodopovidone towelette, the epidural catheter was placed at lumbar level L2-L3, by using the Thuoy 18 G needle and the Braun 20 G catheter; after administration of the test dose with lidocaine 2%/4 mL, the epidural catheter was fixed with the Mepilex Lite patch. Finally, a single-shot spinal anesthesia (8 mg Ropivacaine plus 15 mcg Phentanyl plus 1 mL saline solution—with a total anesthetic volume of 3 mL) was given in the L3/L4 intervertebral space by using the 27 G Transmed Whitacre needle, thus producing a T4 sensory bloc. Meanwhile, 500 mL of saline solution, 500 mL of lactated Ringer’s solution and 10 mg of ephedrine were administered.

Finally, surgery started and, in an effort to minimize any trauma during it, all clinicians avoided inducing pressure or friction on the skin. Surgical and monitoring equipment that came into contact with the patient was well padded or lubricated. A healthy female infant was delivered weighing 1950 g, with Apgar scores of 9 and 10 at one and five minutes, respectively. A Mepilex border flex dressing was set up on the site of incision; a few days later, it was replaced by a Mepilex lite dressing. Throughout the entire procedure, the patient remained hemodynamically stable, awake and cooperative, without experiencing pain or discomfort. Overall, there were no surgical or anesthesiological complications. Blood loss was scarce. 

**Figure 1:**
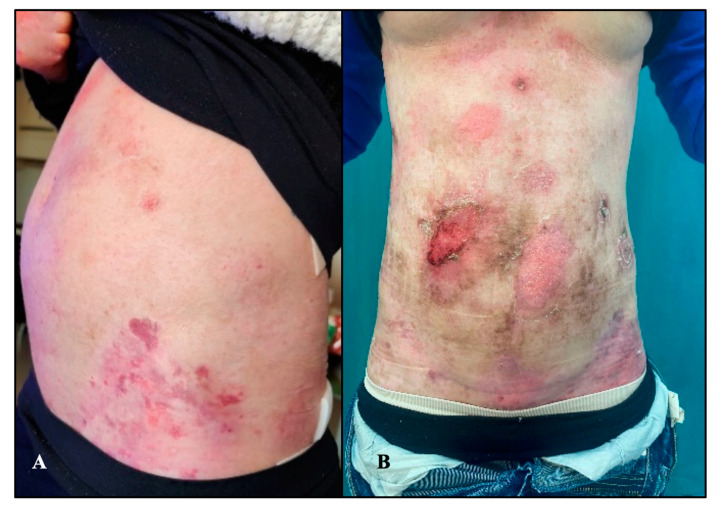
Abdomen macroscopic aspect at 12 weeks of gestation (**A**) and 2 months after childbirth (**B**).

Postoperative analgesia was provided using an epidural elastomer containing Ropivacaine 0.16% and Morphine 3 mg, with a total volume of 240 mL, at an infusion rate of 5 mL/h; in addition, Paracetamol 1gr IV was administered every 8 h for 48 h. The patient was discharged on the third postoperative day, as usual. The scar healed well in a couple of weeks. After childbirth, the itching suddenly disappeared, while the number of skin erosions rose sharply as compared with the pregnancy time, configuring a sort of rebound effect (Figure 1). Breastfeeding was discontinued a few days after delivery because of the appearance of bilateral blisters on the nipples. Overall, both mother and baby are currently doing well.

## 8. Comments and Conclusions

DEB is an extremely rare and disabling inherited genetic skin disease. While a growing number of reports focusing on its pathogenesis, as well as on diagnosis and therapy, are increasingly coming into being, overall research has directed little focus towards pregnancy and childbirth in DEB patients. Worth mentioning, a panel of EB experts has recently come up with a needed guideline on this specific topic [36] that summarizes recommendations achieved by means of a systematic review of the literature and expert consensus. Yet, this guideline focuses on pregnant women suffering from any form of EB, regardless of its severity, hence our idea of realizing an updated synthesis of the existing evidence on obstetrical management specifically focusing on DEB patients while also adding our own experience. Second, but not least, to the best of our knowledge the available literature on this topic, including the guideline in question [36], outlines how EB conditions the management of pregnancy, but not how pregnancy conditions the course of the underlying skin disease. Therefore, this review article also sought to answer the crucial question of whether, and if so in which way, the pregnancy condition may alter the course of DEB itself. Ultimately, by simultaneously bringing together the existing knowledge regarding these two equally fundamental aspects, we deem that our work, together with the sound evidence that already exists, may be extremely helpful for clinicians who have to counsel a patient with DEB who desires a child or is expecting one.

As a rule of thumb, it would be desirable for patients with DEB to undergo genetic counseling early in life to assess the risks of inheritance and consciously plan for a family.

On the whole, pregnancy in DEB patients has proven to be successful, without substantial additional prenatal or postnatal complications as compared to non-affected patients. Additionally, a flaring of the underlying skin disease is rarely demonstrated in the available literature, so avoidance or termination of pregnancy should not be recommended for patients suffering from DEB.

The main complications for pregnant patients with DEB can arise at the time of delivery: this emphasizes the importance of an early MDT counselling to explain to the couple the risks involved in childbirth and to decide early on an appropriate strategy for delivery. Whenever indicated and feasible, vaginal delivery seems to be the safest choice, especially considering the avoidance of airway manipulation. In cases of CS, a risk/benefit assessment should be performed to decide on the most appropriate anesthesia method, opting for RA if possible.

Due to the available negligible and low-quality evidence, further research focusing on pregnancy and delivery in patients with DEB is desirable.

## Data Availability

All data are reported in the text.
